# Misconduct in research: a descriptive survey of attitudes, perceptions and associated factors in a developing country

**DOI:** 10.1186/1472-6939-15-25

**Published:** 2014-03-25

**Authors:** Patrick I Okonta, Theresa Rossouw

**Affiliations:** 1 Department of Obstetrics and Gynaecology, Faculty of Health Sciences, College of Health Sciences, Delta State University, Abraka, Delta State, Nigeria; 2 Department of Family Medicine, Faculty of Health Sciences, University of Pretoria, Pretoria, South Africa

**Keywords:** Research misconduct, Perception, Attitudes, Associated factors

## Abstract

**Background:**

Misconduct in research tarnishes the reputation, credibility and integrity of research institutions. Studies on research or scientific misconduct are still novel in developing countries. In this study, we report on the attitudes, perceptions and factors related to the work environment thought to be associated with research misconduct in a group of researchers in Nigeria - a developing country.

**Method:**

A survey of researchers attending a scientific conference was done using an adapted Scientific Misconduct Questionnaire-Revised (SMQ-R). Initial descriptive analysis of individual items using frequencies and proportions for all quantitative data was performed. Thereafter, Likert scale responses were transformed into dichotomous responses. Fisher exact test was performed for associations as appropriate. A two-tailed p-value of less than 0.05 was accepted as significant.

**Result:**

Half of the respondents (50.4%) were aware of a colleague who had committed misconduct, defined as “non-adherence to rules, regulations, guidelines, and commonly accepted professional codes or norms”. Over 88% of the researchers were concerned about the perceived amount of misconduct prevalent in their institution and 96.2% believed that one or more forms of scientific misconduct had occurred in their workplace. More than half (52.7%) rated the severity of penalties for scientific misconduct in their work environment as low. Furthermore¸ the majority (56.1%) were of the view that the chance of getting caught for scientific misconduct in their work environment was low.

**Conclusion:**

Researchers in Nigeria perceive that scientific misconduct is commonplace in their institutions, but are however worried about the negative effects of scientific misconduct on the credibility of scientific research. We recommend that researchers be empowered with the knowledge and virtues necessary for self-regulation that advance research integrity. Research institutions should however also step into their role of fostering a responsible research ethic and discouraging misconduct.

## Background

Misconduct in research tarnishes the reputation of research institutions and has the potential to diminish the credibility and integrity of research in general. In developed countries, several processes have been put in place in an attempt to protect the credibility of research. Such interventions include regular training in research ethics and responsible conduct of research [1], institutional mechanisms to address research misconduct [2] and the establishment of national bodies that address research misconduct such as the Office of Research Integrity (ORI) in the United States of America [3,4]. However, the extent to which these interventions have been effective in reducing misconduct has not been documented.

Studies on research or scientific misconduct are still novel in the developing world. Fanelli in his article, ‘How Many Scientists Fabricate and Falsify Research? A Systematic Review and Meta-Analysis of Survey Data’, pooled 21 surveys for the systematic review and 18 for the meta-analysis [5]. Fifteen of the studies were from the United States, three from the United Kingdom, two from multi-national samples in developed countries and one study was from Australia. There was no study from Africa; a reflection of the dearth of studies from the region. Similarly, Anah et al., in their literature review of research misconduct in low and medium income countries found no systematic study of research misconduct from these countries [6].

The first study to report on research misconduct and other research wrongdoing in Africa revealed that 68.9% of a group of researchers in Nigeria admitted to having committed at least one of eight listed forms of scientific misconduct [7]. These eight acts of research misconduct were plagiarism; falsifying data; intentional protocol violations related to subject enrolment; intentional protocol violations related to procedures; selective dropping of data from ‘outlier’ cases; falsification of biosketch, resume, or reference list; disagreements about authorship; and pressure from a study sponsor (e.g. pharmaceutical company or device company) to engage in unethical practices. In a later report from the same country, published by Adeleye and Adebamowo, 54.6% of the respondents admitted to at least one form of research wrongdoing [8].

With initial studies thus reporting a high prevalence of research misconduct in Nigeria, it is imperative that factors associated with research misconduct, particularly behavioural attitudes and perceptions of researchers, should be evaluated. Several factors that might contribute to researchers engaging in research misconduct have been suggested and can be broadly categorised as personal, institutional or socio-cultural [9-13]. While these factors are recognised globally, the extent to which they play or interplay in any particular case of research misconduct vary and are probably context specific.

The Federal Ministry of Health of Nigeria published the National Code of Health Research Ethics in 2007 [14]. The Code stipulates guidelines for the ethical conduct of research with emphasis on the protection of human research participants. There is also a National Health Research Ethics Committee (NHREC) whose responsibilities are to: set norms and standards for conducting research on humans and animals, including clinical trials; adjudicate in complaints about the functioning of health research ethics committees; register and audit the activities of health research ethics committees; and recommend to the appropriate regulatory body such disciplinary action as may be prescribed or permissible by law against any person found to be in violation of any norms and standards or guidelines set for the conduct of research. The NHREC is still primarily engrossed with setting up the regulatory framework for institutional ethics committees in the country. Unlike the Office of Research Integrity (ORI) in the United States, the NHREC has as yet no data on research misconduct in the country. Nigeria has no defined or effective national mechanism for responding to research misconduct [6].

In our earlier publication, we had reported on the prevalence of research misconduct and behavioural influences associated with research misconduct [7]. In this report, which is the concluding part of the larger study, we report on the attitudes, perceptions and factors related to the work environment perceived to be associated with research misconduct in a group of researchers in Nigeria.

## Methods

These data are part of a larger study in which 133 researchers completed a self-administered questionnaire during a scientific conference in 2010 [7]. The Scientific Misconduct Questionnaire–Revised (SMQ-R) was adapted for the survey by adding questions that elicited self-reporting of scientific misconduct [15]. In addition, the open-ended part in the original SMQ-R was removed since the questions were not relevant to the Nigerian context. The adapted SMQ-R questionnaire for this survey contained 50 items that elicited responses on the following:

1. Demographic and research experience (questions 1–7)

2. Research and ethical climate at the work environment (questions 8–13)

3. Perceived prevalence of scientific misconduct in the workplace (questions 14–23).

4. Attitude and beliefs about scientific misconduct (questions 24–38)

5. Behavioural influences on scientific misconduct (questions 29–42).

6. Personal involvement in scientific misconduct (questions 43–50)

This paper reports on researchers’ perceptions of the prevalence of scientific misconduct in their workplace and also on their attitudes towards scientific misconduct. Scientific misconduct was defined in this study as “the non-adherence to rules, regulations, guidelines, and commonly accepted professional codes or norms”, since this was the definition used in the original, validated questionnaire [15].

Confidentiality was assured in that researchers were not required to write their names nor that of their institutions on the questionnaire. Furthermore, to assure confidentiality of the group, information about the conference that could lead to possible identification of the group of participants was concealed in this report. Only consenting researchers participated in the survey. Consent was implied by filling the questionnaire after reading the participant information leaflet. The questionnaires were self-administered and upon completion, the questionnaires were dropped in a sealed box at the conference information/welcome area. The research ethics committees of the University of Pretoria, South Africa and the Delta State University Teaching Hospital, Delta State, Nigeria gave ethical approval for the study and the conference organisers gave permission to conduct the study during the conference.

Initial descriptive analysis of individual items using frequencies and proportions for all quantitative data was performed. Thereafter, Likert scale responses relating to research and ethical climate at the workplace were transformed into dichotomous responses: favourable research climate or unfavourable research climate. Numeric scores were given to each Likert response as follows: very low = -2; low = -1; high = +1; and very high = +2. The total score from the 6 items in this section for each respondent ranged from -12 to +12. All negative scores were grouped as ‘unfavourable research climate’, while all positive scores were grouped as ‘favourable research climate’. Fisher exact test was performed for associations as appropriate. A two-tailed p-value of less than 0.05 was accepted as significant.

## Results

We previously reported on the personal involvement of these researchers in scientific misconduct, as well as possible behavioural factors that might have influenced such conduct [7]. We now report on the researchers’ perceptions of research misconduct (defined as non-adherence to rules, regulations, guidelines, and commonly accepted professional codes or norms) and their attitudes and beliefs about research misconduct. The demographic details of the respondents, which had been presented in our earlier published article, is represented here for ease of comprehension.

In this survey of researchers we obtained a response rate of 88.7% (133 out of 150 researchers attending the conference completed the survey). The majority of the researchers (62.4%) worked primarily in academic institutions, while 25.6% worked in public hospitals; 7.3% in private hospitals; 1.5% in the ministry; 0.8% in a research centre; and 2.4% in other sectors. One hundred and twenty one researchers (91.0%) were actively involved in research while only 12 (9%) were not. There were 116 (87.2%) male and 17 (12.8%) female researchers. The median duration of involvement in research was 8 years with an interquartile range of 4–13.5 years. Ninety-two researchers (69.7%) had been involved in research for ten years or less, while 40 (30.3%) had spent more than ten years in research. The median number of publications per researcher was six with an interquartile range of 2–26. Eighty-four researchers (64.1%) had ten or fewer publications while 47 (35.9%) had more than ten publications. The majority of the researchers (77.5%) had attended a lecture, workshop or conference on ethics, but 22.5% had never attended any such training.

### Perception of frequency of occurrence of scientific misconduct in the workplace

When asked to rate how frequently they perceived various acts of scientific misconduct occurred in their workplace, the majority of researchers indicated that plagiarism, falsification of data and selective dropping of data from ‘outlier’ cases occurred ‘occasionally’ (Table 1). The majority of researchers also believed that intentional protocol violations related to subject enrolment, intentional protocol violations related to procedures, falsification of biosketch, resume or reference list and disagreements about authorship occurred ‘seldom’. Pressure from study sponsors to engage in unethical practices was perceived to be the least common type of scientific misconduct in their workplace, while falsification of data was perceived to be the most frequent.

**Table 1:** Percieved occurrence of various aspects of scientific misconduct in the workplace

	**Never**	**Seldom**	**Occasionally**	**Frequently**	*Total no of resp.	**+Non resp.**
Plagiarism	15 (11.5%)	39 (29.7%)	65 (49.6%)	12 (9.2%)	131	2
Falsifying data	12 (9.1%)	40 (30.3%)	62 (47.0%)	18 (13.6%)	132	1
Intentional protocol violations related to subject enrolment	16 (12.5%)	51 (39.8%)	48 (37.5%)	13 (10.2%)	128	5
Intentional protocol violations related to procedures	18 (14.3%)	49 (38.9%)	46 (36.5%)	13 (10.3%)	126	7
Selective dropping of data from ‘outlier’ cases	17 (13.6%)	41 (32.8%)	52 (41.6%)	15 (12.0%)	125	8
Falsification of biosketch, resume, reference list	29 (23%)	52 (41.3%)	34 (27.0%)	11 (8.7%)	126	7
Disagreements about authorship	22 (16.7%)	61(46.2%)	39 (29.5%)	10 (7.6%)	132	1
Pressure from study sponsor (e.g. pharmaceutical company or device company) to engage in unethical practices	48 (38.4%)	42 (33.6%)	31 (24.8%)	4 (3.2%)	125	8

*Total no of resp. = Total number of responses to the question.

+Non resp = Total number of non responses to the question.

On the whole, 128 (96.2%) believed that one or more forms of scientific misconduct had occurred in their workplace while only 5 (3.8%) researchers believed that none of the various types of scientific misconduct ever happened at their workplace. Of these five researchers, four worked in a private hospital and one in an academic institution. Three of the four working in a private hospital had not been actively involved in research.

We further investigated whether researchers’ perceptions of the presence of misconduct at their workplace was associated with researchers having committed acts of scientific misconduct (data on researchers’ self-reported acts of scientific misconduct was presented in our earlier paper). There was no statistically significant relationship: 88 of the 91 researchers (96.7%) who personally admitted committing misconduct and 40 out of 42 researchers (95.2%) who had not committed any acts of scientific misconduct, believed that scientific misconduct had occurred at their workplace, (Fischer exact p-value 0.65).

### Awareness of acts of scientific misconduct in the workplace

Sixty-four researchers (49.6%) were not aware of any particular investigator in their institution who had engaged in scientific misconduct in the past five years, while 33 (25.6%) were aware of one instance; 31 (24%) of 2–5 instances; and one respondent (0.8%) was aware of more than 10 instances. On the whole, 50.4% of the researchers were aware of at least one act of misconduct in their institution. The commonest sources from which researchers became aware of scientific misconduct were from other researchers (36.8%), through personal observation (30.1%), and less commonly through official channels in the institutions (19.5%), from the institution’s ethics committee (16.5%) and from study monitor (8.3%).

### Attitudes and beliefs about scientific misconduct

Over 88% of researchers were concerned about the perceived amount of misconduct prevalent in their institutions and agreed that all professional education programmes should include information about standards of research ethics (Table 2). About 88% of researchers disagreed with the statement: ‘dishonesty and misrepresentation of data are common in society and do not really hurt anybody’. The majority (84.8%) disagreed with the proposition that the responsibility for the scientific integrity of a study lies with the principal investigator only. Interestingly, the majority (83.3%) stated that they did not feel uncomfortable talking with fellow researchers about unethical behaviour if they had to; however, the frequency with which this actually occurred was not assessed.

**Table 2:** Researchers’ attitudes and beliefs about scientific misconduct

	**Agree**	**Disagree**	**Don’t know**	*Total no of resp.	**+Non resp.**
**I am concerned about the amount of misconduct**	**117 (88.6%)**	**4 (3.0%)**	**11 (8.4%)**	**132**	**1**
I think the responsibility for the scientific integrity of a study lies with the principal investigator only	16 (12.2%)	112 (84.8%)	4 (3.0%)	132	1
All professional education programmes should include information about standards of research ethics	128 (98.4%)	1 (0.8%)	1 (0.8%)	130	3
I feel uncomfortable talking with researchers about unethical behaviour	12 (9.3%)	109 (83.8%)	9 (6.9%)	130	3
Dishonesty and misrepresentation of data are common in society and do not really hurt any body	9 (7.0%)	114 (88.3%)	6 (4.7%)	129	4

*Total no of resp. = Total number of responses to the question.

+Non resp = Total number of non responses to the question.

### Rating of work environment in relation to scientific misconduct

Eighty-one percent of the researchers rated the severity of penalties for scientific misconduct in their work environment as low or very low, whereas only 19% rated it as high or very high (Table 3). Furthermore¸ the majority were of the view that the chance of getting caught for scientific misconduct in their work environment was low (56.1%) or very low (19.7%). About 50% of researchers rated the effectiveness of their institution’s rules and procedures for reducing scientific misconduct as low, while another 10% rated it as very low. Only 9% rated the effectiveness of their institution’s rules and procedures for reducing scientific misconduct as very high.

**Table 3:** Researchers’ rating of work environment factors that affect scientific misconduct

	**Very low**	**Low**	**High**	**Very high**	*Total no of resp.	**+ Non-resp.**
Severity of penalties for scientific misconduct	37 (28.3)	69 (52.7%)	18 (13.7%)	7 (5.3%)	131 (100%)	2
Chances of getting caught for scientific misconduct if it occurs	26 (19.7%)	74 (56.1%)	29 (22%)	3 (2.3%)	132 (100%)	1
Researchers’ understanding of rules and procedures related to scientific misconduct	10 (7.6%)	67 (51.1%)	52 (39.8%)	2 (1.5%)	131 (100%)	2
Your own understanding of rules and procedures related to scientific misconduct.	2 (1.5%)	21 (16.1%)	78 (59.5%)	30 (22.9%)	131 (100%)	2
Researchers’ support of rules and procedures related to scientific misconduct	9 (7.0%)	61 (47.3%)	51 (39.5%)	8 (6.2%)	129 (100%)	4
The effectiveness of your institution’s rules and procedures for reducing scientific misconduct	13 (10.0%)	66 (50.8%)	42 (32.3%)	9 (6.9%)	130 (100%)	3

*Total no of resp. = Total number of responses to the question.

+Non resp. = Total number of non responses to the question.

Most researchers rated their own understanding of rules and procedures related to scientific misconduct as high (60%) or very high (23%). In contrast, researchers were of the opinion that few researchers in their own institutions understood these rules. Moreover, they rated fellow researchers’ support of rules and procedures related to scientific misconduct as low. Altogether, using a composite scoring scale, the work environment was considered favourable for preventing scientific misconduct by 53 (40.2%) researchers and unfavourable by 79 (59.8%) researchers.

Analysis did not show a statistically significant relationship between the perceived prevalence of scientific misconduct at the workplace and the work environment (Table 4).

**Table 4:** Association between percieved presence of scientific misconduct in the workplace and the work environment

		**Scientific misconduct at the workplace**		**Test of statistical significance**
		**Present**	**Absent**	
Work environment	Favourable	50	3	Fischer exact = 0.32 95% CI 0.25 – 28.38 p-value = 0.67
	Unfavourable	77	2	
TOTAL		127	5	

## Discussion

Our study revealed that 88% of researchers were concerned about the perceived amount of misconduct prevalent in their institution. Furthermore, an amazingly high number of researchers (96.2%) were of the belief that one or more forms of scientific misconduct had occurred in their workplace. Specifically, 85% and 88% of researchers respectively perceived that plagiarism and falsification of data had occurred in their institution. However, only about 50.4% of the researchers were actually aware of at least one act of misconduct in their institution in the past 5 years. Eighty one percent of researchers rated the severity of penalties for scientific misconduct in their work environment as low or very low and only nine percent rated the effectiveness of their institutions’ rules and procedures for reducing scientific misconduct as very high.

In interpreting our findings we recognise that several factors have to be put into context such as our broad definition of scientific misconduct and our methodology. Also, in comparing our findings with others, it is desirable to select studies with similar methodology as ours such that we compare similar to similar. Fanelli had remarked that the method of questionnaire delivery and, in particular, how the questions are asked, could impact on the results from surveys on scientific misconduct and this must be considered when comparing results [5].

### Perception of frequency of occurrence of scientific misconduct in the workplace

The perception of occurrence of scientific misconduct in the workplace in this study is much higher than any other existing reports on this topic [16,17]. For example, while only 9.1% of our researchers said that falsifying data had never occurred at their workplace, 71.3% of research coordinators interviewed by Pryor et al. in 2005 in the US believed that falsifying data had never occurred at their workplace [16]. Similarly, only 11.5% of our researchers, compared to 66.9% of researchers in the Pryor study, said that plagiarism had never occurred at their workplace. Rankin, in a survey of 88 nursing research coordinators, directors and deans from masters and doctoral level programmes in the US in 1997, reported that 27.2% of the respondents perceived that cheating in data collection had never occurred and 12.5% that plagiarism had never occurred in their institutions [17]. The perception of a higher frequency of acts of misconduct in our study possibly reflects a true difference, since the instruments used in these comparable studies are similar.

A possible consequence of the perception of a high prevalence of misconduct in the workplace is that individuals might lower their moral threshold for committing an offence when it is perceived that everybody else is committing similar offences. Indeed, the relationship between deviant peers and the development of deviant behaviour has been well elucidated by Cohen [18].

An important question raised by these figures is to what extent this perception of high levels of research misconduct reflects reality. Previously published data from our larger study demonstrated that about 69% percent of the respondents admitted to having personally committed at least one of the eight listed forms of misconduct [7]. In a similar study by Adeleye among researchers from the same country, a high proportion of the respondents (54.6%) also admitted to having committed at least one act of research wrongdoing [8]. These two studies underscore the likelihood that the perceptions reported in this study are realistic. It is, however, pertinent to note that both these studies used a broad definition of scientific misconduct.

We did not find any statistically significant association between having committed misconduct and perceiving that misconduct is prevalent in an institution. The lack of any significant association may be due to our use of a broader definition of scientific misconduct, which yielded a very high percentage (96.2%) of perceived misconduct in the workplace. With such an overwhelming imbalance, it will be difficult to establish any association. Nonetheless, it can be argued that this perception of a high prevalence of scientific misconduct might at least partially be based on personal experience and therefore approximate reality.

### Awareness of acts of scientific misconduct in the workplace

Roughly 51% of researchers in our study were aware of investigators in their institution who had engaged in scientific misconduct in the past five years. In comparison, Pryor using the same study instrument, but administered to research coordinators in the US, found that 18.3% of respondents had first-hand knowledge of acts of scientific misconduct in the preceding one year of their study [16]. In Norway, a survey of 189 doctoral students revealed that 28% had heard of cases of unethical scientific behaviour in the 12 months preceding the survey [19]. A study by Swazey et al. showed that only 6 - 9% of students and faculty knew of specific instances were faculty members had plagiarised or falsified data [20]. Fanelli, in the first ever systematic review and meta-analysis, demonstrated a wide range of responses with between 6.2% and 72% of respondents reporting knowledge of various questionable research practices among their colleagues [5].

Our findings seem to mirror the situation reported in some earlier studies (more than a decade ago) in the US and Europe [16,21]. About half of our researchers said that they were aware of at least one case of scientific misconduct occurring during the past five years, while in 1992, Kalichman and Friedman reported that 36% of 549 biomedical trainees at the University of California knew of an instance of scientific misconduct [21] and in 2001, Geggie reported from the United Kingdom that 55.7% of newly appointed consultants had observed some form of misconduct [22].

A possible explanation for this observation could be that the systematic interventions and establishment of institutions to minimize scientific misconduct and encourage credible conduct of research in the US might have impacted positively on the occurrence of scientific misconduct [1,23]. For example, the ORI in the US is the statutory body that oversees and directs the Public Health Service research integrity activities on behalf of the Secretary of Health and Human Services. As stated earlier, in Nigeria, even though a National Health Research Committee (NHREC) exists and has been tasked with various regulatory functions, it is still primarily engrossed with setting up the regulatory framework for institutional ethics committees in the country. Unlike the ORI, the NHREC has as yet no data on research misconduct in the country. Nigeria also has no defined or effective national mechanism for responding to research misconduct [6]. It therefore seems possible that the situation in Nigeria might be lagging behind the developed world scenario.

Another significant finding is that the percentage of researchers (51.4%) who were aware of any particular case of misconduct in their institution in the last five years was considerably lower than the 68.9% (from the same sample in our earlier report) who had admitted to have committed misconduct. Admittedly, the question about awareness of acts of misconduct in their institution was limited to the preceding 5 years, while the question on having ever committed scientific misconduct had no time limit, and this may offer a possible explanation for the observed difference. On the other hand, it may be argued that the observed difference reflects the inability of the institutions to detect research misconduct. Expectedly, scientists who engaged in misconduct would not readily admit to their colleagues (unless anonymously) that they had participated in unethical behaviour.

### Attitudes and beliefs about scientific misconduct

Despite the high level of perceived research misconduct, the majority of researchers reported a positive attitude towards reducing the high level of scientific misconduct in their institutions and were supportive of the idea that all professional programmes should include information on standards of research ethics. The value of education on research ethics as a strategy for reducing scientific misconduct cannot be overemphasised. Vuckovic-Dekic et al. demonstrated that even a short course on research ethics had a positive impact on the attitude of medical researchers towards research misconduct [24].

The demonstrated willingness to address scientific misconduct, coupled with the researchers’ readiness to discuss ethical issues with colleagues, create a welcomed opportunity to plan and implement interventions to minimize research misconduct and enhance the responsible conduct of research.

### Rating of work environment in relation to scientific misconduct

Several authors have highlighted the influence of the work environment on scientific misconduct [2,9,13,25,26]. In this study there was, surprisingly, no statistically significant association between the work environment and the perceived occurrence of scientific misconduct in the workplace. The lack of a statistically significant association may, however, be due to the small sample size or the very high prevalence of perceived misconduct reported in this study. It could also be due to the fact that the questionnaire had been adapted from a different demographic setting or that our approach to the statistical analysis through the conversion of the Likert scale response into a dichotomous response, may have blurred the dividing line.

Nonetheless, several insightful facts did emerge from our analysis of respondents’ rating of their work environment. A small proportion of researchers rated the severity of their institutions’ penalties for scientific misconduct as high; conversely, most researchers rated the chances of getting caught as low. Obviously the work environment depicted by these responses could not possibly provide disincentives for committing scientific misconduct. This inference was confirmed by the fact that the majority of the researchers rated the effectiveness of their institutions’ rules and procedures for reducing scientific misconduct as low. In contrast, the majority of surveyed research coordinators in the US rated the severity of penalties for scientific misconduct as high (75% versus 13.7% in this study), the chances of getting caught as high (68% versus 22%), and the effectiveness of their institutions’ rules and procedures for reducing scientific misconduct as high (87.2% versus 19%) [16].

There is no doubt that academic and research institutions have a big role to play in discouraging scientific misconduct. Institutions should be encouraged to explore the state of research misconduct in their own environments and formulate guidelines for investigating and dealing with suspected cases of scientific misconduct. It might further be appropriate to consider the role of a national body that could coordinate institutional efforts and make resources available to institutions that lack the academic and human resources needed for such an endeavour. We do, however, argue that it is important for institutions to retain autonomy in this regard, given the heterogeneity of research populations and work environments and the importance of respecting the diversity of institutional cultures. The argument for respecting institutional autonomy is further advanced by the disagreement in the literature regarding the appropriate response to research misconduct. While some authors prefer less emphasis on identification of culprits and their punishment, others argue that stiffer punishment and, in fact, criminalization of scientific misconduct is needed to reduce its prevalence [27-29].

We favour an approach of clear, but minimal, institutional intervention with greater emphasis on the role of the individual researcher in regulating his or her own behaviour. Training of researchers should be grounded in a strong foundation of virtue ethics and resources should be made available to enhance knowledge and awareness of various forms of scientific misconduct. There is no doubt that preventing scientific misconduct requires a multi-pronged approach. It is necessary to identify cases of scientific misconduct and prescribe appropriate deterrents, but it is also crucial to implement interventions and preventative measures that could change behaviour associated with scientific misconduct.

### Limitations of our study

Firstly, our definition of scientific misconduct is wider than the definition currently used by the ORI, which restricts it to fabrication, falsification and plagiarism (FFP). The implication of our broader definition is that we recorded a higher prevalence of perceived scientific misconduct. Another implication of our broader definition is that it made comparison with other studies less precise since most studies from developed countries restrict their definition to FFP. Secondly, our sample was a purposive sample of researchers in a particular medical specialty. Therefore, the extent to which we can generalise our findings is limited. Thirdly this study was designed as an exploratory, preliminary, descriptive study and the sample size was not powered to identify associations. Future studies should take cognisance of the high perceived prevalence of research misconduct recorded in our study and use an appropriately powered sample size that would allow for assessment of various associations. Finally, we acknowledge the limitations of our study instrument – the SMQ-R – in exploring certain aspects of research misconduct in an in-depth manner.

## Conclusion

Researchers in Nigeria perceive that scientific misconduct is commonplace in their institutions. They are, however, worried about the negative effects scientific misconduct might have on the credibility of scientific research. Furthermore, they are concerned that their institutions do not have effective mechanisms in place to reduce the occurrence of scientific misconduct. Researchers should be empowered with the knowledge and virtues necessary for self-regulation that advance research integrity. Research institutions should however also step into their role of fostering a responsible research ethic and discouraging misconduct.

## Competing interests

The authors declare that they have no competing interest.

## Authors’ contributions

PO generated the research concept and design, conducted the research and drafted the manuscript. TR made substantial input in refining the research concept and design and revising the draft manuscript. Both authors approved the final version of the manuscript to be published.

## Pre-publication history

The pre-publication history for this paper can be accessed here:

http://www.biomedcentral.com/1472-6939/15/25/prepub

## References

[B1] Department of Health and Social Services Nicholas S ORI Introduction to the responsible conduct of research 2007 Washington: US government printing office 164

[B2] Jeffers BR Whittemore R Research environments that promote integrity Nurs Res 2005 54 1 63 70 15695941 10.1097/00006199-200501000-00009

[B3] Abbrecht P Davidian N Merrill S Price AR The role of the office of research integrity in cancer clinical trials Cancer Treat Res 2007 132 231 239 10.1007/978-0-387-33225-3_13 17305026

[B4] Dahlberg JE Davidian NM Scientific Forensics: How the Office of Research Integrity can Assist Institutional Investigations of Research Misconduct During Oversight Review Sci Eng Ethics 2010 16 4 713 35 10.1007/s11948-010-9208-4 20526693

[B5] Fanelli D How many scientists fabricate and falsify research? A systematic review and meta-analysis of survey data PLoS One 2009 4 5 e5738 10.1371/journal.pone.0005738 19478950 PMC2685008

[B6] Ana J Koehlmoos T Smith R Yan LL Research misconduct in low- and middle-income countries PLoS Med 2013 10 3 e1001315 10.1371/journal.pmed.1001315 23555197 PMC3608538

[B7] Okonta P Rossouw T Prevalence of Scientific Misconduct Among a Group of Researchers in Nigeria 2012 Dev World Bioeth, p. doi:10.1111/j.1471-8847.2012.00339.x 10.1111/j.1471-8847.2012.00339.x PMC3530634 22994914

[B8] Adeleye OA Adebamowo CA Factors associated with research wrongdoing in Nigeria J Empir Res Hum Res Ethics 2012 7 5 15 24 10.1525/jer.2012.7.5.15 23324199 PMC3619034

[B9] Martinson BC Crain AL Anderson MS De Vries R Institutions expectations for researchers' self-funding, federal grant holding, and private industry involvement: manifold drivers of self-interest and researcher behavior Acad Med 2009 84 11 1491 9 10.1097/ACM.0b013e3181bb2ca6 19858802 PMC3071700

[B10] Davis MS The role of culture in research misconduct Account Res 2003 10 3 189 201 14979320 10.1080/714906092

[B11] Hackett EJ A social control perspective on scientific misconduct J Higher Educ 1994 65 3 242 60 10.2307/2943966 11653363

[B12] Davis MS Riske ML Steneck NH, Scheetz MD Preventing scientific misconduct: Insights from convicted offenders Investigating research integrity: Proceedings of the first ORI research conference on research integrity 2002 Rockville MD: Office of research Integrity

[B13] Davis MS Riske-Morris M Diaz SR Causal factors implicated in research misconduct: evidence from ORI case files Sci Eng Ethics 2007 13 4 395 414 10.1007/s11948-007-9045-2 18038194

[B14] Federal Ministry of Health The national code for health research ethics 2007 Abuja: Federal ministry of Health

[B15] Broome ME Pryor E Habermann B Pulley L Kincaid H The Scientific Misconduct Questionnaire–Revised (SMQ-R): validation and psychometric testing Account Res 2005 12 4 263 80 16578917 10.1080/08989620500440253

[B16] Pryor ER Habermann B Broome ME Scientific misconduct from the perspective of research coordinators: a national survey J Med Ethics 2007 33 6 365 9 10.1136/jme.2006.016394 17526690 PMC2598278

[B17] Rankin M Esteves MD Perceptions of scientific misconduct in nursing Nurs Res 1997 46 5 270 6 10.1097/00006199-199709000-00005 9316599

[B18] Cohen AK The Sociology of the Deviant Act: Anomie Theory and Beyond Am Sociol Rev 1965 30 5 14 10.2307/2091770 14247328

[B19] Hofmann B Myhr AI Holm S Scientific dishonesty–a nationwide survey of doctoral students in Norway BMC Med Ethics 2013 14 3 10.1186/1472-6939-14-3 23289954 PMC3545724

[B20] Swazey J Anderson M Louis K Ethical problems in academic research American Scientist 1993 81 542 53

[B21] Kalichman MW Friedman PJ A pilot study of biomedical trainees' perceptions concerning research ethics Acad Med 1992 67 11 769 75 10.1097/00001888-199211000-00015 1418260

[B22] Geggie D A survey of newly appointed consultants' attitudes towards research fraud J Med Ethics 2001 27 5 344 6 11579193 10.1136/jme.27.5.344 PMC1733447

[B23] National Research Council of the National Academics Integrity in scientific research: creating an environment that promotes responsible conduct 2002 Washington: The National Academic press 24967480

[B24] Vuckovic-Dekic L Gavrilovic D Kezic I Bogdanovic G Brkic S Science ethics education part II: changes in attitude toward scientific fraud among medical researchers after a short course in science ethics J BUON 2012 17 2 391 5 22740224

[B25] de Vries R Anderson MS Martinson BC Normal Misbehavior: Scientists Talk about the Ethics of Research J Empir Res Hum Res Ethics 2006 1 1 43 50 10.1525/jer.2006.1.1.43 16810336 PMC1483899

[B26] Franzen M Rodder S Weingart P Fraud: causes and culprits as perceived by science and the media. Institutional changes, rather than individual motivations, encourage misconduct EMBO Rep 2007 8 1 3 7 10.1038/sj.embor.7400884 17203094 PMC1796756

[B27] Sovacool BK Using criminalization and due process to reduce scientific misconduct Am J Bioeth 2005 5 5 W1 7 10.1080/15265160500313242 16179287

[B28] Redman BK Merz JF Sociology. Scientific misconduct: do the punishments fit the crime? Science 2008 321 5890 775 10.1126/science.1158052 18687942

[B29] Solutions, not scapegoats Nature 2008 453 7198 957 10.1038/453957a 18563095

